# The Arabidopsis paralogs, *PUB46* and *PUB48*, encoding U-box E3 ubiquitin ligases, are essential for plant response to drought stress

**DOI:** 10.1186/s12870-016-0963-5

**Published:** 2017-01-11

**Authors:** Guy Adler, Zvia Konrad, Lyad Zamir, Amit Kumar Mishra, Dina Raveh, Dudy Bar-Zvi

**Affiliations:** 1 Department of Life Sciences, Ben-Gurion University of the Negev, 1 Ben-Gurion Blvd, Beer-Sheva, 8410501 Israel; 2 The Doris and Bertie I. Black Center for Bioenergetics in Life Sciences, Ben-Gurion University of the Negev, 1 Ben-Gurion Blvd, Beer-Sheva, 8410501 Israel

## Abstract

**Background:**

Plants respond to abiotic stress on physiological, biochemical and molecular levels. This includes a global change in their cellular proteome achieved by changes in the pattern of their protein synthesis and degradation. The ubiquitin-proteasome system (UPS) is a key player in protein degradation in eukaryotes. Proteins are marked for degradation by the proteasome by coupling short chains of ubiquitin polypeptides in a three-step pathway. The last and regulatory stage is catalyzed by a member of a large family of substrate-specific ubiquitin ligases.

**Results:**

We have identified *AtPUB46* and *AtPUB48—*two paralogous genes that encode ubiquitin ligases (E3s)—to have a role in the plant environmental response. The *AtPUB46, −47,* and −*48* appear as tandem gene copies on chromosome 5, and we present a phylogenetic analysis that traces their evolution from an ancestral *PUB-ARM* gene. Single homozygous T-DNA insertion mutants of *AtPUB46* and *AtPUB48* displayed hypersensitivity to water stress; this was not observed for similar mutants of *AtPUB47*. Although the three genes show a similar spatial expression pattern, the steady state levels of their transcripts are differentially affected by abiotic stresses and plant hormones.

**Conclusions:**

*AtPUB46* and *AtPUB48* encode plant U-Box E3s and are involved in the response to water stress. Our data suggest that despite encoding highly homologous proteins, *AtPUB46* and *AtPUB48* biological activity does not fully overlap.

**Electronic supplementary material:**

The online version of this article (doi:10.1186/s12870-016-0963-5) contains supplementary material, which is available to authorized users.

## Background

Plants respond to abiotic stress with major physiological, biochemical and molecular changes that lead to a new homeostasis. These changes include a global alteration of the plant transcriptome, proteome, and metabolome that result from a new balance between the rates of cellular biosynthesis and degradation activities. Enhanced protein degradation in stress conditions leads to a reduced steady state level of proteins whose optimal levels are much lower in stress conditions than in non-stress conditions. Furthermore, abiotic stress conditions induce the production of reactive oxygen species (ROS) [1] that can also result in irreversibly oxidized proteins and other biologically active polymers that are targeted for degradation [2–4].

The ubiquitin-proteasome system (UPS) is a central eukaryotic system for regulated protein degradation [5, 6]. The proteolytic activity resides in the 26S proteasome present in both the cytoplasm and the nucleus. Proteins are targeted for degradation by the 26S proteasome by covalent attachment of a short chain of ubiquitin molecules [5] performed by a sequence of three enzymes, a ubiquitin activating enzyme (E1), ubiquitin conjugating enzyme (E2), and a ubiquitin ligase (E3) that recognizes the substrate [7]. Thus, it is the E3 that determines whether a given protein will be sent for degradation by the 26S proteasome. Although protein ubiquitylation is mostly associated with degradation, ubiquitylation also plays a role in signaling and modification of protein activities [6] giving the E3s a critical role in cell function.

Over 5% of Arabidopsis genes encode proteins of the UPS with the majority of UPS-related genes (ca 1,700) encoding E3s. E3s can be divided into subfamilies based on their structure and primary amino acid sequence. These include multimeric E3s such as cullin-based E3s, and monomeric E3s such as the RING and U-box protein families [5, 6]. Many UPS genes are induced in response to abiotic stress: these include genes that encode ubiquitin, E2s, E3s and proteasome subunits (reviewed by [8–11]).

Plant U-box (PUB) proteins are a small family of proteins with the U-box motif [10, 12, 13]. The U-box comprises ca. 70 amino acids and resembles a modified RING finger that forms a similar structure stabilized by salt-bridges and hydrogen bonds [14, 15]. PUBs have E3 activity [10, 12, 14]. Of the more than 1000 E3 genes found in Arabidopsis and rice, the PUBs comprise a small family of 64 and 77 genes, respectively [12, 13, 16]. Although PUBs are just a small fraction of the plant E3s, they are far more abundant than in other organisms such as yeast and animals—each of these encodes fewer than ten U-box E3s [10, 12, 13]. PUBs are monomeric: the largest PUB subgroup comprises proteins with a N-terminal U-box followed by Armadillo (ARM) repeats. The ARM motif is a ca. 40 residue long that often appears in tandem non-identical copies in a single PUB and functions in protein-protein interactions [17, 18].

PUB E3s are involved in diverse biological processes such as development, self-incompatibility, and response to hormones. They are widely connected with the plant stress response [8–11, 19, 20]. PUBs play an essential role in drought [21–24], salt stress [21, 25, 26], temperature stress [21, 27], oxidative stress [28], and in the response to phosphate starvation [29].

Given the central role of E3s in selecting specific proteins for degradation, the identification of E3s that are active in the response to a defined stress is an important step towards elucidating the pathways that regulate this response. We therefore initiated a screen of homozygous Arabidopsis E3-T-DNA insertion mutant plants for their response to water stress. The *AtPUB46*- T-DNA insertion mutants were found to be hypersensitive to water stress compared with WT plants. The study was then expanded to the adjacent *AtPUB47* and *AtPUB48* genes, which encode highly homologous proteins. As found for *AtPUB46* mutants, T-DNA insertion mutants of *AtPUB48* displayed increased sensitivity to water stress. On the other hand, sensitivity of *AtPUB47* T-DNA insertion mutants for water stress was not affected. Cell and tissue expression patterns of the *AtPUB46-48* genes are similar; however, we found that they differ in their response to hormones and abiotic stress cues. All three genes encode active E3s as shown using recombinant AtPUB46-48 proteins produced in bacteria. Thus, our results suggest that *AtPUB46* and *AtPUB48* play a role in establishing a new protein homeostasis via the UPS in response to drought.

## Methods

### Plant material

All experiments were carried out with *Arabidopsis thaliana* ecotype Columbia.

#### T-DNA insertion-mutants

T-DNA insertion lines prepared by the Salk Institute Genomic Analysis Laboratory [30] were obtained from the Arabidopsis Resource Center, Columbus Ohio. The lines were: *Atpub46-1*, SALK_096071, T-DNA insert in exon 1, 36 bp from the translation start codon; *Atpub46-2*, SALK_109233, 129 bp upstream of the translation start codon; *Atpub47-1*, SALK_018208, 103 bp into exon 2; *Atpub47-2*, SALK_056774, in exon 1, 64 bp downstream of translation start codon; *Atpub48-1*, SALK_057909, 5’ UTR, 97 bp upstream of the translation start codon; *Atpub48-2*, SALK_086659, exon 1, 285 bp downstream of the translation start codon. All lines were homozygous for T-DNA insertion. Homozygosity was confirmed by PCR analysis.

#### Plant transformation and selection of transgenic plants

Recombinant plasmids were introduced into *Agrobacterium* GV-3101, and the transformed bacteria were used for genetic transformation of Arabidopsis by the floral dip method [31]. Transgenic plants were selected on plates containing 30 μg/ml hygromycin. All experiments were performed on T3 generation homozygous plants containing single-site T-DNA inserts. At least three independent-transformant lines were used for each assay.

### Construct design

#### *Promoter::GUS* constructs

DNA sequences of the respective PUB genes were isolated by PCR using Arabidopsis genomic DNA and promoter-specific DNA primer pairs (Additional file 1: Table S1), and subcloned into the pCAMBIA 1391Z vector upstream of the sequence encoding GUS. Histochemical GUS staining was performed as described [32].

#### Constructs for expression of AtPUBs::eGFP fusion proteins

cDNA amplified DNA fragments were fused to the N-terminus of EGFP in the pSAT4-EGFP-N1 plasmid [33] downstream of the constitutive *CaMV 35S* promoter. The *CaMV 35S:AtPUBs::EGFP* fusion cassette was ligated into pCAMBIA 1302 replacing the *CaMV 35S:6xHis-GFP* sequence originally found in this vector.

#### Constructs for expressing recombinant proteins in *E. coli*

The DNA sequences encoding full-length Arabidopsis proteins were prepared by PCR using cDNA from Arabidopsis seedlings as a template, and primer sets described in Additional file 1: Table S1. The resulting protein-encoding sequences were sub-cloned, in-frame, into the indicated bacterial expression vectors. The following constructs for the expression of recombinant Arabidopsis proteins in *E. coli* were made: UBE8 and UBE10 in pHIS-Parallel2 and Arabidopsis PUB46, PUP47, and PUB48 in pGST-Parallel2 [34]. Plasmid Ube1/PET21d, expressing 6xHis-UBE1 was purchased from Addgene (http://www.addgene.org/34965/). All constructs were sequenced to verify that they are in frame and that there are no mutations in the amplified sequences.

### Plant growth and Stress application

Seeds were surface sterilized and cold treated before sowing as described [32]. Plants were grown in Petri dishes containing half strength Murashige and Skoog (0.5 x MS) nutrient solution mix [35] supplemented with 0.5% sucrose and 0.6% agarose, or in pots containing planting mix at 22–25 °C and 50% humidity in a 12 h light/12 h dark regime. Where indicated, plates also contained hormones, antibiotics, or abiotic-stress agents. Application of stress and hormones to two-week old seedlings was performed by the transfer of plate-grown seedlings to Whatman No 1 filter paper soaked in 0.5 x MS and with the indicated concentration of the hormone/stress-inducing chemical.

#### Seed germination and cotyledon greening assay

Surface-sterilized cold-treated seeds were sown on Petri plates containing 0.5 x MS, 0.7% agar, and when applied, the indicated MV, NaCl, or mannitol. Plates were incubated at 22 °C in a 16 h light/8 h dark regime. Green seedlings were scored 5 days later.

#### Drought tolerance

Plants were grown for 3 weeks in pots containing equal amounts of potting mix under non-stressed conditions. Water was then withheld and plant wilting and drying was followed daily.

#### Water loss

Rosettes of one-month old plants were cut and placed with their abaxial side on weigh boats. Samples were weighed immediately after cutting, and in ~10 min intervals. Data from each plant was normalized to its weight at time 0.

#### Photosynthetic efficiency

Photosynthetic efficiency of photosystem II was assayed using MINI-PAM-II fluorometer (Walz GmbH, Effeltrich, Germany). Plants were dark-adapted for 30 min. Each genotype contained 8 soil grown plants. Chlorophyl fluorescence emitted from rosette leaves of controlled and stressed plants was assayed in dark-adapted plants (F_o_), and maximum fluorescence values were measured following an intensed light flash (F_m_). The F_v_/F_m_ values representing photosynthetic efficiency were calculated by (F_m_-F_o_)/F_m._

### Transcript levels

RNA isolation, cDNA synthesis, primer design and RT-qPCR assays for determining relative steady state transcript levels were performed as previously described [36]. Primers are listed in Additional file 1: Table S1.

### Recombinant protein expression

*E.coli* BL21 (DE3) pLYS cells were transformed with the plasmids described above. Cultures were grown at 37 °C to OD_600_ = 0.5. Cultures were then cooled to 16 °C, and expression of recombinant proteins was induced by adding 0.5 mM IPTG. Bacterial cells were harvested after 16 h at 16 °C and suspended in the buffer recommended by the manufacturers of the applicable affinity chromatography resins. Cells were sonicated, and the homogenates were cleared by centrifugation followed by supernatant loading onto the appropriate column. His-tagged proteins were purified on Ni-Charged resin (GenScript, New Jersey, USA), GST-tagged proteins on glutathione resin (GenScript, New Jersey, USA), and MBP-tagged proteins on amylose resin (New England BioLabs, Massachusetts, USA) according to protocols recommended by the manufacturers. Purified proteins were concentrated and chromatography elution buffers were exchanged with phosphate buffered saline (PBS) using Vivaspin 6 centrifugation ultrafilters (Sartorius, Germany). Protein aliquots were stored at −75 °C.

### In vitro ubiquitylation assay

An in vitro ubiquitylation assay was performed using a modification of a previously described assay [37]. The 30 μl reaction mixtures contained 5 μg of ubiquitin (Sigma-Aldrich, USA), 100 ng of the his-tagged human E1 Ube1, 500 ng each of the indicated his-tagged Arabidopsis E2 and GST-tagged E3 in a reaction buffer containing 25 mM Tris–HCl, pH 7.5, 1 mM MgCl_2_, 1 mM ATP, and 0.5 mM DTT. Reactions were incubated at 30 °C for 2 h and terminated by adding SDS gel sample buffer and heating at 95 °C for 5 min. Proteins were resolved by SDS-PAGE, electroblotted onto nitrocellulose membranes, and probed by western blot analysis using an anti-ubiquitin antibody.

### Statistical analyses

Each experiment was performed with at least three biological replicates with more than 50 plants in each treatment. The results are presented as mean ± SE [calculated using SPSS software version 18 (SPSS Inc, Chicago, IL). Differences between groups were analyzed by Tukey’s HSD post-hoc test (*P* ≥ 0.05).

## Results and discussion

### Gene organization of At5G18320, At5G18330 and At5G18340 and domain organization of the AtPUB46, AtPUB47 and AtPUB48 proteins they encode

**Fig. 1:**
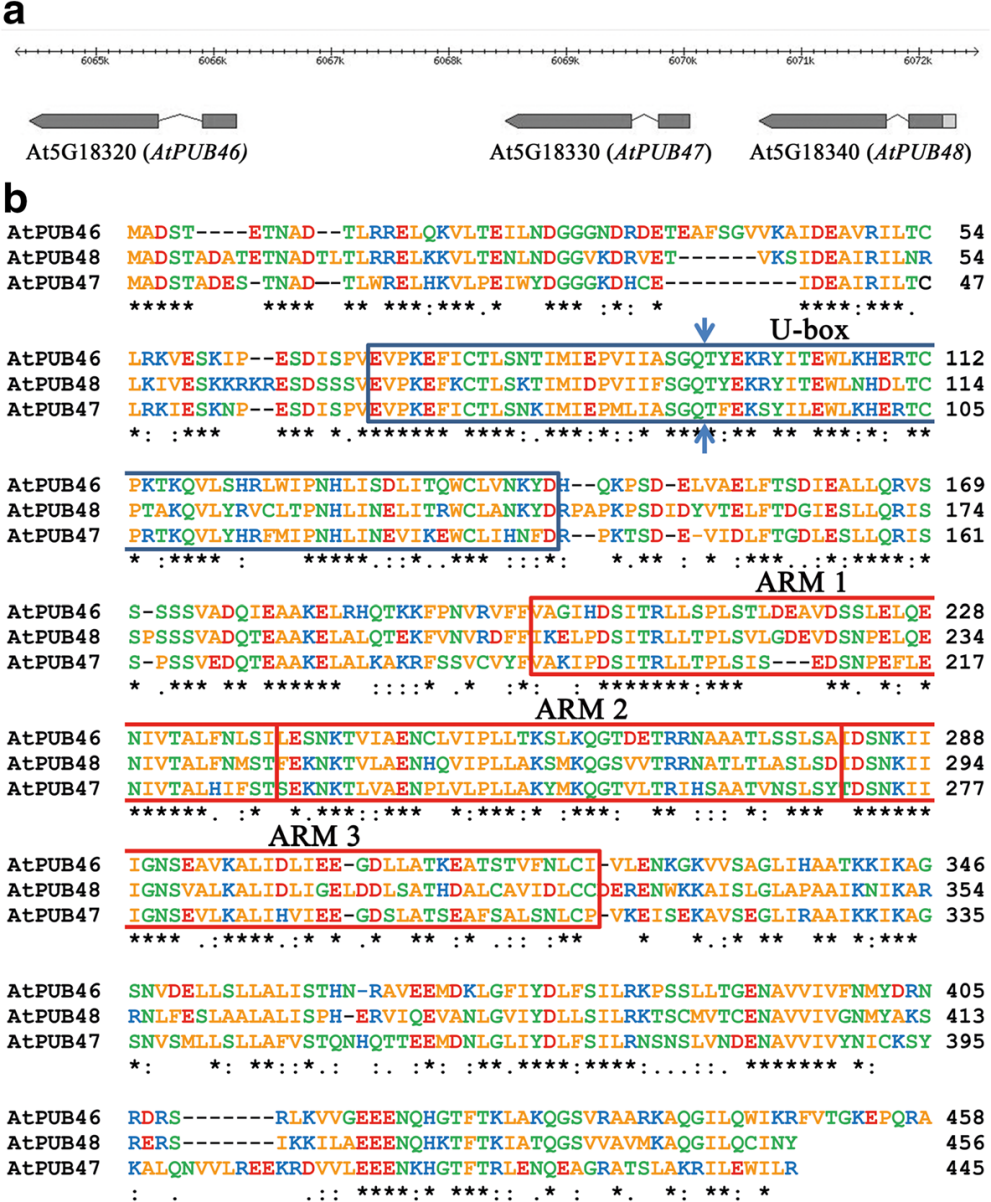
Genome organization of the *AtPUB46, AtPUB47* and *AtPub48* genes and the proteins they encode. **a** Genome organization of Arabidopsis chromosome 5 loci At5G18320, AtG18330, and At5G18340 that encode the *AtPUB46*, *AtPUB47,* and *AtPUB48* genes, respectively. Exons are shown as wide and introns as narrow lines. Arrows mark gene orientation. **b** Amino acid sequence alignment and domain structure of AtPUB46, AtPUB47, and AtPUB48: *blue* (positivly charged), *red* (negatively charged), *green* (polar) and *orange* (non-polar) residues. U-box is shown in a *blue frame*, ARM motifs are marked by *red frames*. Arrows indicate the position corresponding to the exon-exon borders

We screened Arabidopsis E3 T-DNA insertion mutants for altered tolerance to water stress and found a homozygous T-DNA insertion mutant of *AtPUB46* with enhanced sensitivity to water stress. This gene is a member of a cluster of 3 loci (At5G18320, At5G18330 and At5G18340) on the upper arm of chromosome 5 that encode highly homologous U-box protein ligases (*AtPUB46* to *AtPUB48*, respectively, Fig. 1a). These three genes are in the same orientation and are encoded by the lower DNA strand. A phylogenetic tree of the 64 PUB proteins encoded by Arabidopsis indicated that AtPUB46, AtPUB47, and AtPUB48 form a small distinct cluster [13, 20]. Amino acid alignment shows that AtPUB46 shares high similarity with both AtPUB47 and AtPUB48 (63–65% identity and 73–75% similarity), whereas AtPUB47 and AtPUB48 are somewhat less similar to one another (55% identity and 65% similarity). Each protein has a U-box close to the N-terminus followed by three copies of an ARM motif (ARM1-3; Fig. 1b), which was first identified in Drosophila Armadillo protein and shown to function in protein-protein interactions [38]. The corresponding ARM motifs of the three paralogs are very similar with sequence identities of 60–71% for each of the corresponding ARM1, ARM2, and ARM3 motifs. On the other hand, there is much lower homology between the three consecutive ARM motifs (ARM1, ARM2 and ARM3) of each protein. The high degree of similarity between the corresponding ARM1-3 motifs of the three AtPUB46-48 proteins is evidence for gene duplication of a primordial PUB-ARM1-3 gene. Gene duplication is common in Arabidopsis with 15–20% of the genome comprising tandem-arrayed genes (TAG) [39, 40]. Only 17% of these duplication events have resulted in tri-genes of which a large proportion are expressed in response to abiotic stresses [41]. Furthermore, the genes encoding AtPUB46-48 have a single intron that intervenes between the codons that encode residues Q and T within the PUB motif. These correspond to residues 96 and 97 in AtPUB46, respectively (Fig. 1b, arrows). Interestingly, only five of the 64 Arabidopsis *PUB* genes (*PUB9, 24, 46, 47, and 48*) have a single intron. *AtPUB9* on chromosome 3 has an intron at the same position as in *AtPUB46-48* (see Fig. 1b, arrows). Interestingly, the AtPUB9 protein has the highest similarity to AtPUB46-48 on the phylogenetic tree constructed based on the U-box domain of AtPUBs [13] suggesting shared ancestry. The fifth intron-containing PUB gene, *AtPUB24,* has its intron at a different position and is less similar to *AtPUB9* and *AtPUB46-48*. Thus, the first gene duplication must have given rise to *AtPUB9* and the ancestor of the tri-genes. Subsequent gene multiplication leading to *AtPUB46, AtPUB47 and AtPUB48* probably resulted from gene conversion or unequal crossing over. This was certainly not from retrotransposition because the three genes have retained their intron. Furthermore, we can exclude whole genome duplication since the tri-genes are located in tandem on the same chromosome. This reconstructed evolutionary history is similar to that described for the Arabidopsis *MYB* genes [42]. We therefore examined all three genes and the proteins they encode.

### Tissue specific expression of *AtPUB46*, *AtPUB47* and *AtPUB48*

**Fig. 2:**
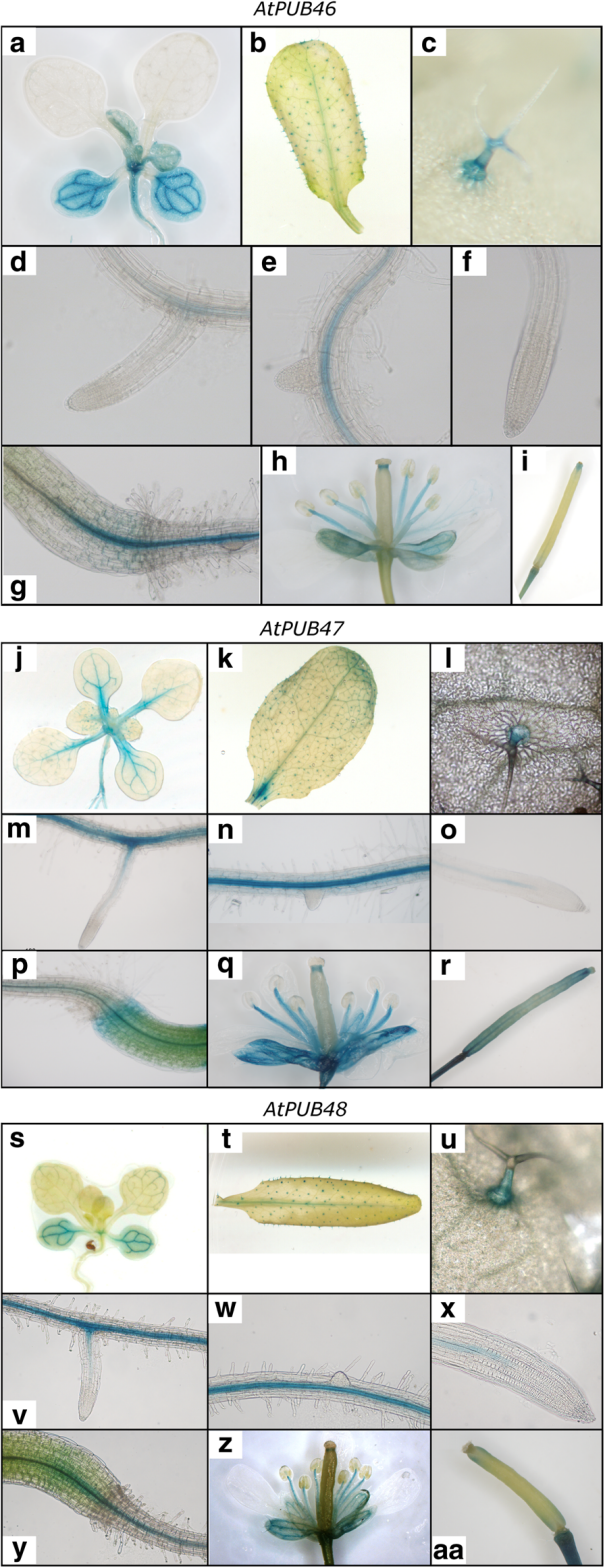
Expression pattern of the *AtPUB46-48* promoters. Arabidopsis plants expressing the *GUS* reporter gene driven by the *AtPUB46* (**a**-**i**) *AtPUB47* (**j**-**r**) or *AtPUB48* (**s**-**aa**) promoters were stained for GUS activity. (**a**, **j**, **s**), 2 week old seedlings; (**b**, **k**, **t**), rosette leaves of mature plants; (**c**, **l**, **u**), trichomes; (**d**, **e**, **m**, **n**, **v**, **w**), primary roots, root hairs and developing lateral roots; (**f**, **o**, **x**), root tips; (**g**, **p**, **y**) shoot-root transition zone; (**h**, **q**, **z**) flowers; (**I**, **r**, **aa**), siliques. At least 3 independent lines were assayed for each construct

To study cell and tissue specificity of expression of the three paralogs, we transformed Arabidopsis plants with *promoter::GUS* constructs. Promoter sequences comprised the entire region upstream of the ATG translation start codon to the STOP codon of the adjacent upstream gene yielding 1440, 597 and 1613 bp promoter sequences for *AtPUB46, AtPUB47,* and *AtPUB48*, respectively. At least three homozygous transformants were selected for each construct, and promoter activity was determined using GUS histological staining. The expression patterns directed by each of the three promoters were very similar: they express in the vascular systems of leaves (Fig. 2a, j, s), roots (Fig. 2d-f, m-o, v-x), stem-root transition zone (Fig. 2g, p, y) and in trichomes (Fig. 2b, c, k, l, t, u). The genes also express in reproductive organs: sepals, short styles, stamen filaments (Fig. 2h, q, z), and receptacles at both the flower and fruit stages (Fig. 2h, i, q, r, z, aa). No staining was observed in root tips (Fig. 2f, o, x) or in petals and anthers (Fig. 2h, q, z). Some differences in expression patterns were observed: *AtPUB46* and *AtPUB48* but not *AtPUB47* express in cotyledon parenchyma (Fig. 2a, j, s). The *AtPUB46* is highly expressed in cotyledons and developing leaves and at lower levels in fully expanded leaves (Fig. 2a). *AtPUB47* is highly expressed in petioles (Fig. 2j), and *AtPUB48* is highly expressed in cotyledons and at a low level in leaves at all stages of development (Fig. 2s).

### Steady state transcript levels of *AtPUB46*, *AtPUB47* and *AtPUB48* in roots and shoots determined by RT-qPCR

The *AtPUB46-48* genes are highly homologous paralogues, and thus the proteins encoded by these genes may function in a redundant fashion. Gene specific activity thus may result from differential expression patterns of each gene. Therefore, we analyzed the steady state levels of *AtPUB46-48* transcripts in root and shoots under different treatments.

**Fig. 3:**
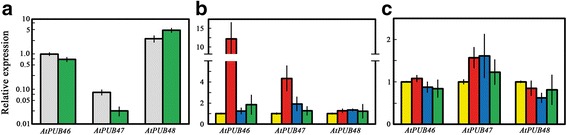
Expression levels of the *AtPUB46-48* genes in roots and shoots of 10 d old seedlings untreated or treated with plant hormones. RNA was extracted from roots and shoots, cDNA was prepared and transcripts levels of the indicated genes were analyzed by RT-qPCR. **a** Root (*gray*) and shoot (*green*); transcript levels of all genes were normalized to that of *AtPUB46* in the roots. **b**, **c** of seedlings were incubated for 6 h with 0.5 x MS without supplements (*yellow*), or supplemented with 10 μM each of IAA (*red*), zeatin (*blue*) or ABA (*green*). Transcript levels were assayed in roots (**b**) and shoots (**c**). Data shown are average ± SE. Expression levels of each gene in the respective organ of non-treated plants were defined as 1

Transcripts of *AtPUB48* are the most abundant—2- and 5-fold higher than those of *AtPUB46* in roots and shoots, respectively (Fig. 3a). In contrast, the levels of AtPUB47 transcripts are at least ten-fold lower than of both the other genes. The *AtPUB46* and *AtPUB47* mRNAs are more abundant in the roots with a shoot/root expression ratio of 0.7 and 0.3, respectively. *AtPUB48* shows higher expression in shoots with a shoot/root ration of 1.8 (Fig. 3a). The low *AtPUB47* expression may be due to its short promoter sequence—only 597 bp to the next upstream gene (Fig. 1a).

### Hormone regulation of expression of *AtPUB46, AtPUB47 and AtPUB48*

Plant hormones play a central role in the response to water- and salt-stress, and ABA is the major hormone involved [43]. We thus assayed the effects of exogenous ABA, auxin, and cytokinin on the expression of *AtPUB46-48* in roots and shoots. The UPS system is known to be involved in hormonal signaling [8–11, 19, 20]. Thus, we assayed the steady state levels of the genes studied here in response to hormone treatment. The three genes respond differentially to application of plant hormones: expression of *AtPUB46* and *AtPUB47* in the roots was markedly enhanced by auxin and to a lesser extent by ABA and cytokinin (Fig. 3b). In contrast, steady state levels of *AtPUB48* in root transcripts were not affected by the hormone treatments. Furthermore, the steady state levels of transcripts of these three genes in the shoots were only marginally affected by all three hormones: *AtPUB47* mRNA levels were moderately induced by auxin and cytokinin, and *AtPUB48* transcript levels were slightly reduced by cytokinin (Fig. 3c). Our data suggest that *AtPUB46* and *AtPUB47* may also be involved in modulation of target proteins whose activity/steady state levels are affected by auxin. Although auxin is mainly associated with plant growth and development, recent studies show that it also plays a role in the response to drought. For example, the rice gene *TLD1*/*OsGH3.13* that encodes indole-3-acetic acid (IAA)-amido synthetase enhanced the expression of *LEA* (late embryogenesis abundant) genes, which correlated with the increased drought tolerance of rice seedlings [44]. We thus suggest that AtPUB46 and AtPUB47 may also be involved in the response to auxin.

### Transcript levels of *AtPUB46, AtPUB47 and AtPUB48* are differentially affected by abiotic stresses

Although salt stress generally causes osmotic-stress as well as ion toxicity, transcriptome analysis of plants exposed to salt- and osmotic-stresses revealed that most genes show a differential response to these two stresses [45]. We therefore measured steady state transcript levels of *AtPUB46-48* in the roots and shoots of seedlings exposed to different abiotic stresses.

#### Salt stress

**Fig. 4:**
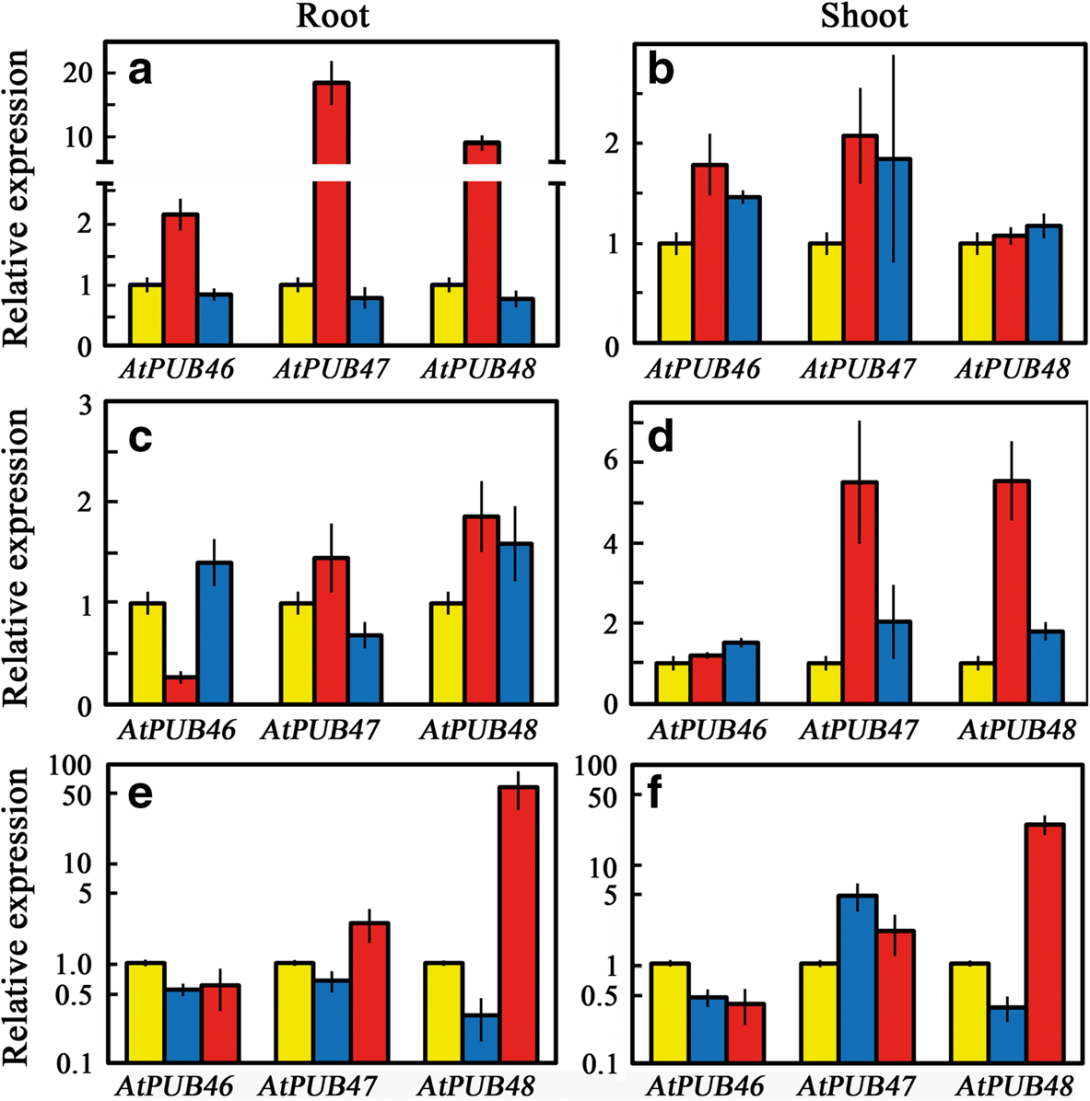
Expression levels of *AtPUB46-AtPUB48* genes in response to abiotic stress analyzed by RT-qPCR. Ten day old seedlings were exposed to the following treatments: (**a**, **b**) salt- and osmotic stress: control (*yellow*); 0.2 M NaCl (*red*); 0.4 M mannitol (*blue*) for 6 h. **c** & **d**, oxidative stress: control (*yellow*); 100 mM H_2_O_2_ (*red*); 1 μM methyl viologen (*blue*) for 3 h in the light. **e**, **f** temperature stress: 25 °C (*yellow*); 3 h at 4 °C (*blue*); 15 min at 45 °C (*red*). Data shown are average ± SE

NaCl treatment evoked a differential response: increased transcript levels in the roots of all three genes but only elevated *AtPUB46* and *AtPUB47* in the shoots (Fig. 4a, b).

#### Osmotic stress

Mannitol did not affect transcript levels of the three studied *AtPUB* genes in the roots, but did enhance the levels of *AtPUB46* and *AtPUB47* in the shoots (Fig. 4a, b).

#### Oxidative stress

The H_2_O_2_-treated seedlings displayed elevated mRNA levels of *AtPUB47* and *AtPUB48* in both roots and shoots; *AtPUB46* showed reduced transcript levels of *AtPUB46* in the roots and unchanged levels in the shoots (Fig. 4c, d). Similarly, methyl viologen (MV) enhanced the expression of *AtPUB47* and *AtPUB48* in the shoots (Fig. 4d) and to a lesser extent of *AtPUB46* in roots and shoots and of *AtPUB48* in the roots. Arabidopsis plants exposed to H_2_O_2_ or MV show very different gene profiles for each treatment [46, 47]. In agreement, the steady-state levels of *AtPUB46-48* transcripts were induced more by NaCl than by a similar osmotic stress administrated by mannitol (Fig. 4a, b).

#### Heat stress

The *AtPUB48* was markedly induced in roots and shoots following heat exposure (Fig. 4e, f); *AtPUB47* was also induced but to a lesser extent. On the other hand, the expression of *AtPUB46* was reduced in all vegetative parts following heat treatment (Fig. 4e, f). *AtPUB48* and *AtPUB46* transcript levels were reduced by low temperatures in both roots and shoots, whereas cold treatment increased the levels of *AtPUB47* transcripts in the shoots (Fig. 4e, f).

Heat shock transcription factors (HSFs) are major players in the induction of heat-responsive genes [48]. Analysis of the putative promoter sequence of the *AtPUB48* gene for HSF binding sites (HSE) (http://bioinformatics.psb.ugent.be/webtools/plantcare/html/) revealed a putative HSE element: CTCGAAGTTTCTAG in the 5' UTR, which is −65 to −53 bases upstream of the translation ATG codon. This matches the HSE consensus sequence CTNGAANNTTCNAG first identified in Drosophila and shown to function in plants [49].

Thus, although the three genes are expressed for the most part in the same cell types (Fig. 2), their differential response to plant hormones and abiotic stresses (Figs. 3 and 4) and the identification of a HSE uniquely in the promoter of *AtPUB48* indicates that their activity does not entirely overlap.

### *AtPUB46*, *AtPUB47* and *AtPUB48* encode catalytically active E3s

**Fig. 5:**
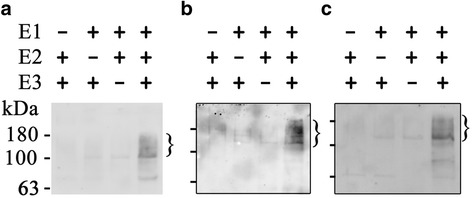
AtPUB46-48 possesses E3 activity. Self-ubiquitylation of each E3 was assayed in vitro using purified recombinant proteins. Uniquitylated protein (marked by } sign) were detected by western blot using anti-ubiquitin antiserum. **a** AtPUB46; **b** AtPUB47; **c** AtPUB48. **a** & **b** had the E2, AtPUBC10 (At5G53300); **c** had AtUBC8 (At5G41700)

Bioinformatics analysis indicated that AtPUB46, AtPUB47, and AtPUB48 are PUB-ARM E3s ([10, 12–14, 16] and www.Arabidopsis.org). To test this directly we produced recombinant GST-tagged AtPUBs, His-tagged human E1, and His-tagged Arabidopsis E2 in *E. coli*. Recombinant proteins were purified by affinity chromatography, and E3 activity was assayed by auto-ubiquitylation of the E3. High MW ubiquitylated proteins were observed in reaction mixtures that contained E1, E2, and E3 (Fig. 5), indicating that all three recombinant AtPUB proteins possess E3 activity. Lower levels of protein polyubiquitylation could also be detected in reaction mixes containing two of the three enzymes in this short metabolic pathway. Similar partial ubiquitylation activities were reported over 30 years ago where mixes containing 2 of the E1, E2 and E3 enzymes yielded 20–44% of the activity obtained in a full reaction mix [50]. Polyubiquitylation by E1 + E3 without E2 or by E1 + E2 without E3 was also recently reported [51–53].

### Homozygous *Atpub46* and *Atpub48* T-DNA insertion mutants are hypersensitive to water stress

**Fig. 6:**
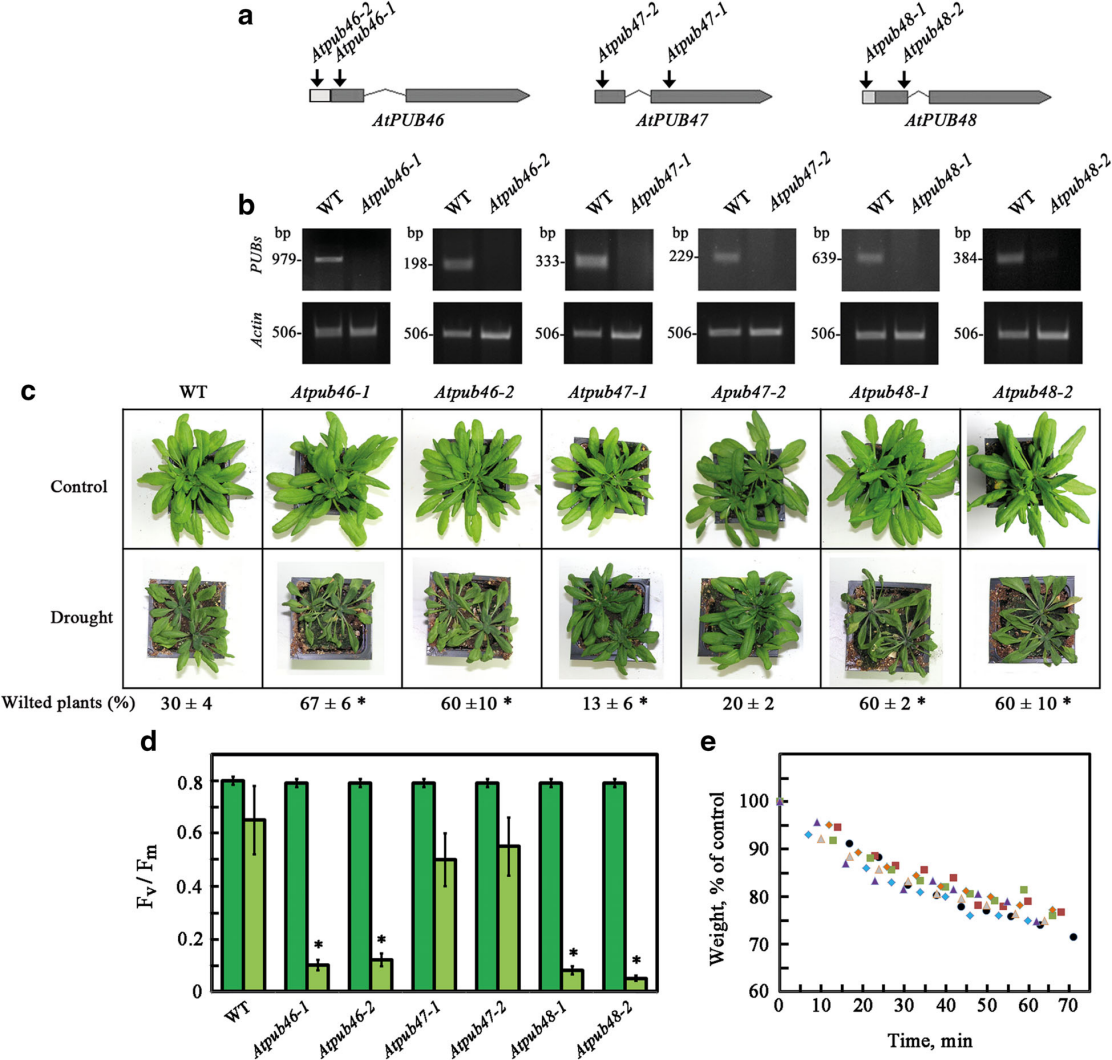
Water stress performance of pot-grown *Atpub46-48 mutant* plants. **a** Location of the T-DNA insertion in the studied mutants. Exons and introns are shown as wide and narrow lines, respectively. Arrows mark gene orientation. The location of the T-DNA insertions in the mutant lines used in this study are marked by arrows. **b** Upper panels: analysis of the indicated *AtPUB46-48* genes in 1-week old wild type (WT) and the indicated mutants using gene-specific primers that anneal to sequences on both sides of the T-DNA insertions. Lower panels: the expression of *ACTIN2* was used as an internal control. **c**, **d** Water stress performance of *Atpub46-48* mutant plants*.* Plants were grown in pots for 3 (**d**) or 4 (**c**) weeks and then water was withheld from drought treated plants. **c**, Plants were photographed after 10 days. **d** Photosynthetic efficiency was assayed 20 days after water withheld. **e** Detached rosettes of 1 month old pot grown plants were assayed for water loss. Data shown are average ± SE. Statistically significant changes from WT plants (*P* < 0.05) are marked with asterisks

Our original screen for Arabidopsis homozygous T-DNA insertion mutant plants for altered water stress sensitivity identified the T-DNA insertion mutant *Atpub46*-plants (SALK_096071) as hypersensitive to water stress. To extend this observation to the adjacent E3s, we used six T-DNA insertion lines—two for each of the *AtPUB46-48* genes: *Atpub46-1* (SALK_096071), *Atpub46-2* (SALK 109233), *Atpub47-1* (SALK_018208), *Atpub47-2* (SALK_056774), *Atpub48-1* (SALK_086659) and *Atpub48-2* (SALK_057909) (Fig. 6a). The T-DNA insertion sites in the *Atpub46-1, Atpub47-2* and *Atpub48-2* mutants disrupt the sequences that encode the U-box suggesting that these are loss-of-function mutants. The *Atpub47-1* mutant has a T-DNA insert in exon2 and probably encodes the U-box domain. It may act as a dominant positive mutant. In the *Atpub46-2* and *Atpub48-1* mutants, the T-DNA is inserted in the 5' UTR. The T-DNA insertions in the 5’ UTRs have been proposed to result from reduced gene expression [54] and also significantly affect protein translation [55]. Thus, *Atpub46-2* and *Atpub48-1* can be regarded as knockdown mutants [56]. The RT-PCR analyses of these mutants confirmed that their transcripts are affected by the T-DNA insertions at the respective sites (Fig. 6b).

Pot-grown plants of these T-DNA insertion mutants were assayed for water stress sensitivity (Fig. 6c). All four *Atpub46* and *Atpub48* mutants were hypersensitive to a lack of water (Fig. 6c). *The Atpub47-2* mutant did not significantly alter plant survival under water deficit, whereas the *Atpub47-1* mutant displayed slightly increased tolerance (Fig. 6c). These differences may be attributed to the location of the T-DNA insertion (above), which may allow expression of the U-box domain in the *Atpub47-1* mutant but not in the *Atpub47-2* mutant.

We have measured chlorophyll fluorescence during the process of water deprivation. A decrease in chlorophyll fluorescence was used for quantitative assessment of drought survival in agreement with previous reports showing a sharp decrease in Fv/Fm values only when Arabidopsis plants were irreversibly affected by drought [57]. Figure 6d shows that *Atpub48* and *Atpub46* mutants completely lost their photosynthetic potential at the same time where type plants and Atpub47 mutants were only slightly affected. This confirms the wilting experiments shown in Fig. 6c. The reduction in chlorophyll fluorescence were seen when leaves of the mutants became necrotic.

Water loss experiments resulted in detached rosettes and showed that the water-loss rates in the wild type and the mutant plants were similar (Fig. 6e). This suggests that that the hyper-drought sensitivity of *Atpub46* and *Atpub48* mutants do not result from impaired stomata function.

**Fig. 7:**
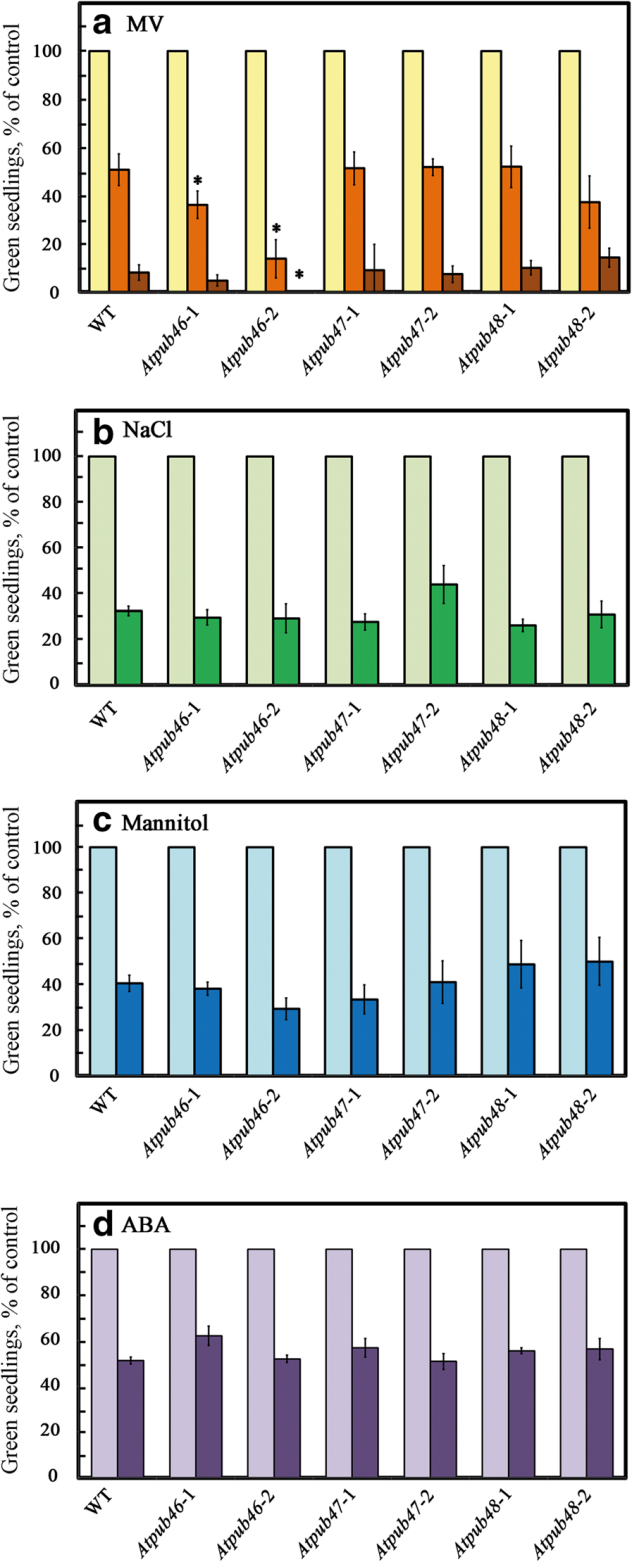
Effects of oxidative, salt and osmotic stresses on seedling germination. Surface sterilized cold treated seeds of the indicated plant lines were plated on agar media containing 0.5 x MS, 0.5% sucrose (control) supplemented with: **a** methyl viologen (MV) at 0 (*yellow bars*), 0.5 μM (*orange bars*) or 1 μM (*brown bars*); **b** NaCl at 0 (*light green*) or 150 mM (*green*) NaCl; **c** mannitol at 0 (*light blue*) or 300 mM (*blue*); **d** ABA at 0 (*light purple*) or 1 μM (*purple*). *Green seedlings* were scored 5 (**a**-**c**) or 6 (**d**) days later. Data shown are average ± SE. Statistically significant changes from WT plants (*P* < 0.05) are marked with asterisks

The above mutants were assayed for germination and seedling establishment under control conditions and abiotic stresses (Fig. 7). When germinated on standard medium, seeds of all tested lines (WT and mutants) were fully germinated suggesting that the mutations do not affect seed viability or germination. The *Atpub46-1* and *Atpub46-2* mutants were hypersensitive to MV-promoted oxidative stress, whereas the extent of inhibition of seedling greening of the *Atpub47* and *Atpub48* mutants did not differ or was only marginally different from that of WT seedlings, respectively (Fig. 7a). Germination in the presence of NaCl or mannitol was not affected in any of the tested mutants (Fig. 7b, c). Moreover, the inhibition of seed germination of the mutants by ABA was not significantly different than that of WT (Fig. 7d). Although mannitol treatment is often used as an osmotic stressor, exposing plants to osmotic shock via high mannitol concentrations may differ from gradually increased water stress induced by withholding water from plants growing in soil [58, 59].

The water stress hypersensitivity observed for T-DNA insertion mutants of the *AtPUB46 and AtPUB48* genes suggests that the biological activities of these genes do not fully overlap. On the other hand, the sensitivity of the *Atpub47* mutants to water stress was probably not altered because their expression levels are negligible compared with those of *AtPUB46* and *AtPUB48* (Fig. 3a) or because of functional redundancy with other E3(s). Gene redundancy is observed when the respective gene products share activity as well as temporal and cell-type expression. Thus, expression in different cell types at different developmental stages or in response to different cues is expected to appear as non-redundant even if the protein activity is identical. Gene families are very common in plants, and the resulting functional redundancy means that most single loss-of-function mutants do not have a phenotype [60].

### *AtPUB46* and *AtPUB48* have a distinct response to water stress compared with other *PUB* genes involved in the response to drought

A number of *PUB* genes are involved in the response to drought: *CaPUB1* from the hot pepper *Capsicum annuum* as well as *AtPUB18, AtPU19, AtPUB22* and *AtPUB23* [21–24]. However, the role of these genes in the response to water stress is opposite that of *AtPUB46* and *AtPUB48*. The *Atpub19*, *Atpub22* and *Atpub23* mutants showed enhanced tolerance to drought; in contrast, *Atpub46* and *Atpub48* mutant plants were hypersensitive to water stress (Fig. 6). These results suggest that at least some of the protein targets of AtPUB46 and AtPUB48 E3 activity are degraded in water stress conditions. Our data suggests that protein targets of AtPUB46 and AtPUB48 are likely to negatively regulate the water stress response because their expected accumulation in *Atpub46* and *Atpub48* mutants decreases plants tolerance to water stress.

## Conclusions

The paralogous *AtPUB46-48* genes located in tandem on Arabidopsis chromosome 5 resulted from gene duplication. We showed that these genes have a unique function in response to water stress because single homozygous mutants of *AtPUB46* and *AtPUB48* are hypersensitive to water stress. Our results suggest that the biological activities of *AtPUB46-48* genes are at least partially gene specific. This specificity may result from differential spatial and/or temporal expression and from possible differences in their substrate specificity. Protein targets of AtPUB46 and AtPUB48 are likely to negatively regulate the water stress response. The Identification of these target proteins will enhance our understanding of their role in the control of specific protein levels under non-stress and stress conditions.

## Additional file

Additional file 1: Table S1. List of primers used in this study. (DOCX 18 kb)

## Abbreviations

ABA: abscisic acid
ARM: Armadillo domain
E1: ubiquitin activating enzyme
E2: ubiquitin conjugating enzyme
E3: ubiquitin ligase
IAA: Indole-3-acetic acid
PUB: Plant U-box
UPS: ubiquitin proteasome system
