# Supplementary material for: The Arabidopsis paralogs, PUB46 and PUB48, encoding U-box E3 ubiquitin ligases, are essential for plant response to drought stress
Source: BMC Plant Biol. 2017 Jan 11;17:8. doi: 10.1186/s12870-016-0963-5 (PMC5225562; doi:10.1186/s12870-016-0963-5)
Supplement: Additional file 1: Table S1. — List of primers used in this study. (DOCX 18 kb) [file 12870_2016_963_MOESM1_ESM.docx]

Table S1. List of primers used in this study

| *Used for | Reverse primer | Forward primer | Primer pair | No. |
| --- | --- | --- | --- | --- |
| OE plant | TATGTCGACGGATCGGTATATATGTATCATGGAG | GACTCTAGAACATATTTGAGTTTCTTCGCTTC | PUB46 coding | 1 |
| OE plant | TATGTCGACAAATCTCATCTCCACTAGTG | GACTCTAGACTTGGCTTCTCTCTACATTCC | PUB47 coding | 2 |
| OE plant | AGTGGATCCGTATCAAATGTAACTTTGCTGAAAC | GACTCTAGAGAAGCAGAGGAAGAAGAATAACTG | PUB48 coding | 3 |
| PR-GUS | TAGGATCCTTCAGCCGGAGAATTCTCG | AGTCTGCAGGACCTGTAAATATGAGTCGCAC | PUB46 promoter | 4 |
| PR-GUS | GATGGATCCTTCAGCCGGAGATTTCTCT | TACTGCAGGGTATTCCCTGCTGGTATG | PUB47 promoter | 5 |
| PR-GUS | GATGGATCCTTTCAGCCCGGAGATTTCTC | TACTGCAGGTGTAGGTTAGGCACCTTATTG | UB48 promoter | 6 |
| *E.coli* | ataGAATTCatATGGCGTCGAAGCGGATC | aatGAATTCtaATGGCTTCGAAACGGATCTT | UBC8 cloning in pHIS- Parallel2 FOR | 7 |
| *E. coli* | attCTCGAGTTAGCCCATGGCATACTTC | ataGAATTCatATGGCGTCGAAGCGGATC | UBC10 cloning in pHIS- Parallel2 | **8** |
| *E. coli* | AT GAATTCTCATGCCCTCTGTGGCTCTTTAC | ATCTGCAGATGGCGGATTCGACGGAAAC | PUB46 cloning in pRSET-C | **9** |
| *E. coil* | TTAGTGATTGTCATCAGTTTTAGG | CTGCAGATGTCTCTCCAACGACCC | HYH cloning in pRSET C | **10** |
| *E. coli* | TCCCAGTCACGACGTTGTAAAACGACGGCCAGTGCCTTAGTGATTGTCATCAGTTTTAGG | GATCgatACGACCGAAAACCTGTATTTTCAGGGCGCCAtaATGTCTCTCCAACGACCCAA | HYHin pMBP-Parallel (Gibson cloning) | **11** |
| qPCR | TGGTTTGGTATCCAAAGACGGT | TTGCTTCTGGGCAGACGTATG | qPCR PUB46 | 12 |
| qPCR | AAAGGACTTGTTTGGTTCTAGGGC | TGGCCAGACTTTTGAAAAGAGC | qPCR PUB47 | 13 |
| qPCR | TCATACGTCTGCCCAGAAAATATGA | GAATCGAAGAAGCGGAAGCG | qPCR PUB48 | 14 |
| qPCR | AGGCCAACACAATAGGATCGA | AAGCAAGCCTACGCTCTGGA | qPCR 18S rRNA | 15 |
| RT-PCR | CGCATGAATCAAACCCGCTG | GCGGCGGGAGTTACAGAAA | *Atpub46-1* analysis | 16 |
| RT-PCR | CGCAGAGTATCGGCGTTTGT | AAGATTAAAGAAAAAGAGCCAACTG | *Atpub46-2* analysis | 17 |
| RT-PCR | TTGGCCGATCAAAGTTGTGAATTAG | ACGGAGGAGGCAAAGATCACT | *Atpub47-1* analysis | **18** |
| RT-PCR | TCAAAAGTCTGGCCAGAAGCGATG | TGTGGCGGGAGTTACACAAG | *Atpub47-2* analysis | **19** |
| RT-PCR | CGAGTCTACCTCATCGCCCA | ACACTGAGGCGGGAGTTGAA | *Atpub48-1* analysis | **20** |
| RT-PCR | ACAGGATCGATCATGATCGTC | GAAGAATAACTGATTTACATAACAGTTATTTC | *Atpub48-2* analysis | **21** |
| RT-PCR | TCATACGGTCAGCGATACCTGAG | TGGTCGTACAACCGGTATTGTGC | Actin2 | **22** |

*OE-plant, constructs for the production of overexpressing plant.

PR-GUS, Promoter::GUS constructs

*E. coli,* expression of recombinant protein in *E. coli*

qPCR, primers for RT-qPCR analysis

RT-PCR, expression study of the T-DNA insertion mutants
